# Immune System, Inflammation and Autoantigens in Wet Age-Related Macular Degeneration: Pathological Significance and Therapeutic Importance

**DOI:** 10.3390/life13122236

**Published:** 2023-11-21

**Authors:** Sreeraj Kuruppilakath Manikandan, Ann Logan, Marc Cerrada-Gimenez, Laurence Fitzhenry, Lee Coffey, Simon Kaja, Sweta Rani

**Affiliations:** 1 Ocular Therapeutics Research Group, Pharmaceutical and Molecular Biotechnology Research Centre, South East Technological University, Waterford Campus, X91 K0EK Waterford, Ireland; sreeraj.kuruppilakathmanikandan@postgrad.wit.ie (S.K.M.); laurence.fitzhenry@setu.ie (L.F.);; 2 Department of Biomedical Sciences, Warwick Medical School, University of Warwick, Coventry CV4 7HL, UK; ann.logan@warwick.ac.uk; 3 Experimentica Ltd., 70211 Kuopio, Finland; 4 Departments of Ophthalmology, Molecular Pharmacology & Neuroscience, Loyola University Chicago, Maywood, IL 60153, USA

**Keywords:** injury, autoantigens, protective autoimmunity, inflammation, aging, neovascularization, macular degeneration

## Abstract

Wet age-related macular degeneration (wAMD) is a chronic inflammation-associated neurodegenerative disease affecting the posterior part of the eye in the aging population. Aging results in the reduced functionality of cells and tissues, including the cells of the retina. Initiators of a chronic inflammatory and pathologic state in wAMD may be a result of the accumulation of inevitable metabolic injuries associated with the maintenance of tissue homeostasis from a young age to over 50. Apart from this, risk factors like smoking, genetic predisposition, and failure to repair the injuries that occur, alongside attempts to rescue the hypoxic outer retina may also contribute to the pathogenesis. Aging of the immune system (immunosenescence) and a compromised outer blood retinal barrier (BRB) result in the exposure of the privileged milieu of the retina to the systemic immune system, further increasing the severity of the disease. When immune-privileged sites like the retina are under pathological stress, certain age- and disease-related conditions may necessitate assistance from cells distant from the resident ones to help restore the functionality of the tissue. As a necessary part of tissue repair, inflammation is a major response to disease and recruits immune cells to the site of damage. We suspect that the specific reparative inflammatory responses are controlled by an autoantigen-T cell-mediated mechanism, a process that may be hindered in wAMD.

## 1. Introduction

**Figure 1:**
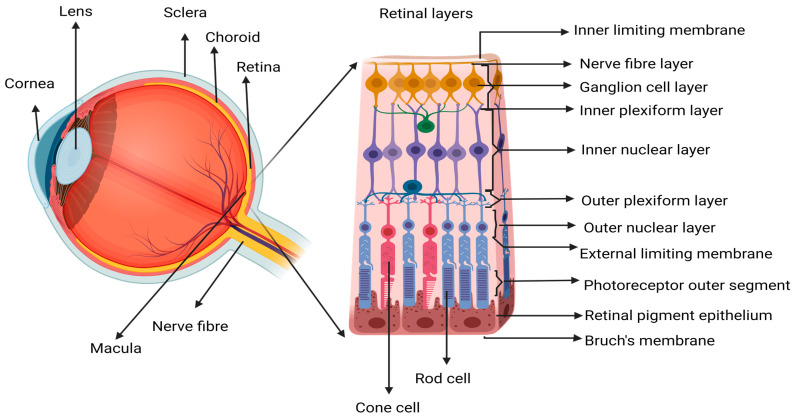
Schematic representation of the retinal structure. Showing an enlarged view of the neurosensory retina, where the central area is called the macular region, which is mostly composed of cone photoreceptor (PR) cells. The loss of macula PR cells affects the central vision since the cones are responsible for activities dependent on visual acuity like reading, driving, writing, and color recognition. Image created in Biorender.com.

One of the leading causes of vision loss in older people is age-related macular degeneration (AMD). It is projected that the number of people suffering from AMD will reach 288 million by the year 2040 [1,2,3]. The increasing number of elderly people diagnosed with AMD is a socioeconomic challenge and a challenge to healthcare, as it affects quality of life and independence in this patient population. AMD affects mainly the choroid (including the choriocapillaris), Bruch’s membrane (BM), retinal pigment epithelium (RPE), and photoreceptors (PR) located central to the neural retina (Figure 1). The progressive nature of the disease often leads to a sequence of pathological stages, beginning with early-stage dry AMD (dAMD) pigmentary changes, accumulation of metabolic waste/deposits, and death of PR and RPE, which can progress to chronic disease states such as wAMD, geographic atrophy (GA), or subretinal fibrosis [4,5,6,7,8,9,10,11,12,13].

Currently, there is no cure for early or dAMD and associated conditions; however, recently, pegcetacoplan (Syfovre) and avacincaptad pegol (Izervay) administered intravitreally were approved by the FDA for the treatment of GA [14,15]. The mainstay therapy for wAMD is anti-vascular endothelial growth factor (anti-VEGF) therapy. Other potential therapeutic targets could be placental growth factor (PIGF) and angiopoietin 2, and both play a central role in neovascularization [16,17,18,19,20,21,22]. Irrespective of the available treatment options, there is a significant treatment burden associated with intravitreal therapy (IVT), including the need for frequent injections, variable vision outcomes, and of course the risks associated with injections, such as retinal detachment, infective endophthalmitis, and drug-induced uveitis [23,24].

The major risk factors of wAMD include increasing age combined with lifestyle risk factors such as the Western diet and smoking in individuals with genetic predispositions such as polymorphisms of the complement system, as revealed by genome-wide association studies (GWAS) [25]. The early pathological sign of AMD is drusen and the increase in the size of RPE drusen deposits in the basal and linear areas of RPE is an early indicator of AMD development risk [26,27,28,29]. Impaired autophagy and metabolic clearance by the RPE cells contribute to the development of drusen. Local immune cells are the initial responders to any infection or injury in the retina. When the cycle of retinal injury and repair continues unabated, the resident immune cells require support from the systemic immune cells in a similar manner to that reported in neurodegenerative diseases by Schwartz and Shechter in 2010 [30]. If the retinal injuries/damage become persistent such that the resident immune cells become exhausted and sustained systemic support is required, this eventually may result in a chronic inflammatory response [31,32]. This paper introduces a perspective about the inflammatory mechanisms that are beneficial in promoting the repair of damaged tissue when mediated through autoantigen-activated T cells.

### 1.1. Inflammation, Immune Response, and Repair in Wet AMD

Inflammation is a natural response that is initiated against a stimulus potentially dangerous to cells, and its purpose is the protection of cells, tissues, or organs from the spread of the incipient infection/damage [33,34]. It involves a coordinated cascade of events like vasoconstriction, vasodilation, release of histamines/kinins/chemokines, migration of immune cells, endothelial attachment, extravasation, chemotaxis, phagocytosis, and elimination of the causative organism/cell/agent. The response subsides in time in a period called the resolution of inflammation. The subsidence of the inflammatory process then leads to the restoration of affected/injured tissue structure and function through repair or regeneration [34]. The resolution of inflammation is of paramount importance when it comes to the restoration of tissue structure and function, and although sustained inflammation is not usually the primary causative factor of chronic disease, its significance to disease evolution cannot be ruled out [34]. The transition of a pro-inflammatory to an anti-inflammatory (wound healing) state is a complex switching process which is dependent on the timely action of key molecules associated with the process [31,33]. For example, the switching of prostaglandin stimulation in the pro-inflammatory state to lipoxin stimulation in the anti-inflammatory state is crucial for the resolution of inflammation [31]. The same cytokines involved in initiating a pro-inflammatory milieu may later switch to an anti-inflammatory influence, acting as a double-edged sword in the process of inflammation resolution. Importantly, the autocrine loop of immune cells, regulatory events, cell signaling, and biochemical events all need to maintain homeostatic function to effectively carry out the events leading to tissue restoration.

Inflammation is a crucial factor in the pathogenesis of AMD [4,23,29,32,35]. Key elements of the inflammatory response are induced when the pattern recognition receptors (PRRs) face perceived danger. The immune system is alerted by endogenous factors called alarmins, which communicate with the immune cells to induce immune responses. These alarm responses are detected by toll-like receptors (TLRs), which also bind to exogenous antigens called pathogen-associated molecular patterns (PAMPs) to elicit a cascade of pro-inflammatory responses [36]. AMD pathogenesis is generally regarded as a possible interplay of genetic factors, hypoxia, and metabolic irregularities. For example, the accumulation of oxidative stressors in tissues that occurs as part of aging activates the PRRs, initiating pro-inflammatory responses as a secondary response to accumulating tissue damage [32]. The initial low levels of tissue stress induce para-inflammation, an intermediate state that is contrary to the basal homeostatic state, rather than a classic inflammatory response [31]. Para-inflammation is the small-scale supply of immune cells to assist the resolution of the stressful situation and to restore functionality and homeostasis in the affected tissue when a local immune response alone cannot handle the situation [32]. Para-inflammation responses are dysregulated [35] when local immune cells are exhausted [32] and thereby contribute to the development of chronic inflammatory conditions associated with many progressive diseases [31].

Prior to disease initiation, para-inflammatory responses support tissue homeostasis through a small-scale supply of immune cells to the damage or injured sites [31,32]. As tissue injuries/damage accumulate over time, these para-inflammatory responses eventually convert to a chronic inflammatory state, as a more robust inflammatory response is initiated in the damaged sites. At some stage, the retina may secrete autoantigens as a mediator for communicating with the adaptive immune system. When this communication between the autoantigen and the adaptive immune system is appropriately controlled, a favorable inflammatory response is initiated; however, detrimental autoreactivity can happen when this control is lost. Many factors can change the course of safe autoreactivity. In a weakened immune system, where autoantigens cannot be processed in a timely manner or provide nutrient perfusion, oxygen, and immune cells to the injured site, this can complicate the timing of events during repair and can elicit a pathogenic state that progressively worsens. The significance of autoantigens present during different stages of the repair process or disease progression requires a deeper exploration as their purpose may not be the same at different stages.

In AMD, the persistence of para-inflammatory states may lead to chronic inflammation and contribute to movement to later stages of the disease [32]. When there is already a chronic inflammatory state existing in tissues, the accumulation of further insults over time could invite additional acute inflammatory responses that drive new adverse tissue responses including soft drusen development, RPE/PR atrophy, and oxidative damage, all acting as antigenic stimuli [8,32,37,38]. A simultaneous occurrence of acute and chronic inflammation with continuous metabolic and oxidative stress can create a persistent hyperinflammatory state culminating in a pathologic state. Dysregulated immune responses will eventually lead to incomplete repair, scar tissue formation, and a failure to restore the functionality of the original tissue, leading to persistent disease states.

Resident retina immune cells, notably microglia, also contribute to the transition from a transient inflammatory state to a persistent disease state. Microglia are activated and migrate to the sub-retinal space during injury. The microglial migration from inner to outer retina is compensated by the recruitment of systemic monocytes, which become macrophages after entering the retina from the circulation [39]. These macrophages, together with the retinal microglia, play a major role in the para-inflammation response, which involves the clearance of the sub-retinal space from debris and restoration of tissue homeostasis [29,31]. Activation of the adaptive immune system is required to recruit immune cells and initiate crosstalk to assist in tissue repair. Crosstalk occurs via the release of autoantigens and antigen presentation to T cells. Here, the adaptive immune system exerts protective effects and aids in repair, in part due to the exquisite specificity that is required to supplement the innate response in mediating and resolving inflammation [40] when resident retinal immune cells have become dysregulated. Differences in the extent of the protective adaptive response may explain which inflammatory environment favors AMD. We believe that focusing on the role of the adaptive immune system in AMD pathology can shed light on potential therapeutic targets as well as provide a better understanding of the role of inflammation and immune system responses in the pathogenesis of AMD.

This is based on the working hypothesis that AMD progression can be attributed to a persistent local inflammatory state in the retina causing PR and RPE insult. Cell debris and aggregated proteins accumulating in the retina activate the resident microglia to phagocytose these unwanted inclusions. Upon phagocytosing the debris, the microglia secrete pro-inflammatory cytokines and toxic compounds like reactive oxygen species (ROS), in a similar manner to the CNS disease conditions proposed by Schwartz and Shechter [30]. If prolonged/uncontrolled microglial activation exists, the survival of nearby uncompromised cells may become affected by the secretion of these toxic agents and further activation of microglia. Autoantigens may be released by the retinal cells at this stage (something similar to the ‘equilibrium stage’ proposed by Schwartz and Raposo in 2010 and 2014) [30,41] to bring help from the systemic immune system in an attempt to maintain homeostasis. These autoantigens presented to T cells induce an autoimmunity response, which is protective to the injured area [41]. As a part of this T cell response, monocytes are recruited to the retina and differentiate into macrophages displaying a similar morphology to microglia. These monocyte-derived macrophages assist in clearing the debris and suppress microglial activation by secreting anti-inflammatory cytokines and growth factors, so that the inflammatory response moves towards resolution.

The outer retina, where PRs are located, is avascular and receives metabolic support from the choroid. In pathogenic states, the required transport and clearance mechanisms of nutrients and metabolic waste may be interrupted in the retina. RPE, the macrophages and monocytes in the neighboring tissue or the choroid itself start giving signals to develop new blood vessels to supply nutrients and more immune cells to the hypoxic retina. When comparing this mechanism to AMD, it is a normal part of the inflammatory and proliferative stage of wound healing that is clearly represented in wAMD [42,43], and a response which at some point turns pathogenic.

A reason for this may be the result of insufficient inflammation and macrophage trafficking at the injury site required to clean or make the site debris-free for the maturation of angiogenic vessels. For the proper maturation of blood vessels and new tissue formation to occur in disease-compromised tissue, retinal immune cells need to efficiently complete the autocrine loop of recruiting more macrophages to the injury site. Signals from macrophages and RPE stimulate monocytes to differentiate to a dendritic-like phenotype and then migrate to the lymph nodes with the antigens acquired from the injured area where they present them to the adaptive immune system. Such activity requires a robust inflammatory response to recruit monocytes to respond to retinal antigens and interact with the adaptive immune system. An appropriate tissue response is dependent on the further involvement of the adaptive immune system to help and responsibly execute the necessary steps that eventually lead to new tissue formation. This is achieved either by RPE de-differentiation or migration to restore lost cells, or through the differentiation of bone-marrow-derived/adult stem cells. Gradually, when the repair process is completed, the involution of blood vessels occurs, and scar tissue will be formed without having a major effect on the integrity of retinal layers.

#### Immunosenescence

A possible reason for the dysfunctional immune system in AMD could be immunosenescence, i.e., the aged and exhausted local immune cells in the retina that are unable to communicate with the systemic immune system. Immunosenescence could also play a role in the functional changes arising in T cell-mediated pathways. For example, CD28 receptor activation stimulates T cells to produce cytokines, chemokines, and signals for their expansion as well as their differentiation [44]. T cells start losing the expression of CD28 with age, leading to a reduced overall immune response and this is also found to have an association with AMD [45].

T cells isolated from AMD patients showed a higher expression of the immunosenescent marker CD56 and loss of the CD28 receptor, indicating a reduced protective T cell response [46,47]. The over-expression of immunosenescent markers could generate functional differences in the normal state as well, for example, changes in the expression of the programmed cell death protein-1 (PD-1) and T cell immunoglobulin domain 3 (TIM-3) markers are associated with T cell exhaustion, and regulating these markers has been found to restore T cell effector function [48]. Cross-recognition of antigens by receptors is also a factor in the T cell functional difference, as it could replace the required response. However, senescence is inevitable for T cells, like all aging cells, so cellular irregularities associated with aging can be a result of failure of the endogenous repair process, as well as due to cellular susceptibility to disease or pathologic condition.

A well-orchestrated, adequately replenished inflammatory response is required in such a pathological scenario to deliver protection from further damage and to ensure that functionality and structure are restored in the affected tissues [40]. When the exhausted immune cells and overwhelmed local reparative functions that occur in AMD fail to mediate a successful controlled inflammatory response, this culminates in advancing stages of AMD and progressive loss of vision [49,50].

The loss of effector function, immunological tolerance, and antigen-specific immunosuppressive functions of T cells contribute to the progression of neurodegenerative diseases like AMD. It is not just single factor/receptor/cytokine dysregulation that is involved in the disease etiology, but multifactorial events, implicating an exhausted immune system as a result of aging. Indeed, T cells are required for disease modification, but again the context is important [51]. Context determines whether T cells initiate physiological or pathological responses, and appropriate T cell subsets are required to orchestrate the processes of tissue repair and regeneration that determine disease progression. A further important factor to be considered is whether the immune pathology is the key factor in determining the progression of a disease like AMD, or whether it is the decrease in the abnormal deposits (drusen) that are required for disease modification.

### 1.2. The Concept of Autoimmunity in AMD

Autoimmunity is defined as a response of the immune system that is mounted against its own/host cells, tissues, proteins, or other normal components of the body. These ‘self-proteins’ or ‘autoantigens’ are recognized by antibodies (autoantibodies) that orchestrate a response to maintain homeostasis at the site of antigen release. Under normal circumstances, autoantibodies are generated in order to destroy cancerous cells, cells infected by microorganisms, injured cells, cells that are dying, or for a specific purpose, that needs further investigation. The reactivity of these autoantibodies is highly dependent on the microenvironment, disease conditions, and the genetic traits of the individual [52].

The autoimmunity associated with a break in anergy/tolerance, in other words in a system that has ‘gone out of control’, induces the possibility of detrimental autoreactive T cell expansion [53]. Autoreactive T cells are normally beneficial when activated through antigen presentation or through growth factor-expressing resident microglia in the CNS [54]. Sometimes, an immune response may contribute to an ongoing pathology by being reactive against self-proteins and cells, inducing inflammatory responses that can have collateral damage to neighboring tissues [55]. While this may be an attempt to aid recovery, a badly timed intervention or an inadequate response may fail to generate the required cell activity (phenotype) [55]. Another possible scenario is the transition of inflammation and immune responses to the later stages of repair with incomplete cellular responses in the initial stages. What is necessary in AMD is an immune response towards the RPE that will facilitate repair in the initial stages of injury. If this does not happen successfully, a recurring immune activity occurs at the site (RPE) and the insult persists. This continuous process of unsuccessful recovery by the immune system weakens the efficiency of RPE in carrying out normal functions like eliminating the metabolic waste, resulting in the formation of debris within the RPE–BM–choroid interface [28,56,57].

Now, despite the pre-existing immune activity to repair retinal damage, these debris generate another set of immune responses designed to prevent them from accumulating or acting as antigenic stimuli. These are, in turn, self-reactive responses when taken over by antigen-presenting cells (APCs), as they break down the peptides in drusen and present them to T cells. These peptides can be perceived as a threat to the eye by the immune system that can then mount a response for clearing them through an antibody response to these proteins, and thus collateral damage is initiated. As a result, the escalating immune response affects normal retinal functioning, where damage may happen to essential proteins, exacerbating the damage in the RPE [58].

The presence of autoantigens and autoantibodies in aging and in AMD eyes is reported in several scientific studies [9,59,60,61,62,63,64,65,66,67,68,69,70]. Although these studies report the involvement of autoantigens and autoantibodies in both conditions, the exact mechanism of their origin and their role in disease pathogenesis is unclear, even after many years of research [52,59]. In AMD, the existing inflammatory response might not be sufficient to attenuate the ongoing pathology, resulting in a compromise of the repair function of RPE cells. We speculate that the release of autoantigens by the retina in AMD is initiated to facilitate repair by inducing more inflammation and immune cell recruitment to the damage site. Under normal physiology, the autoantigens are necessary for retinal functioning. In disease, these autoantigens might be released due to the breakdown of homeostasis in the retina in order to enhance autoreactivity (Table 1). However, this controlled and beneficial autoimmune response can sometimes become detrimental, based on the nature of the disease and whether the response intensity and timing are suitably regulated [54].

**Figure 2:**
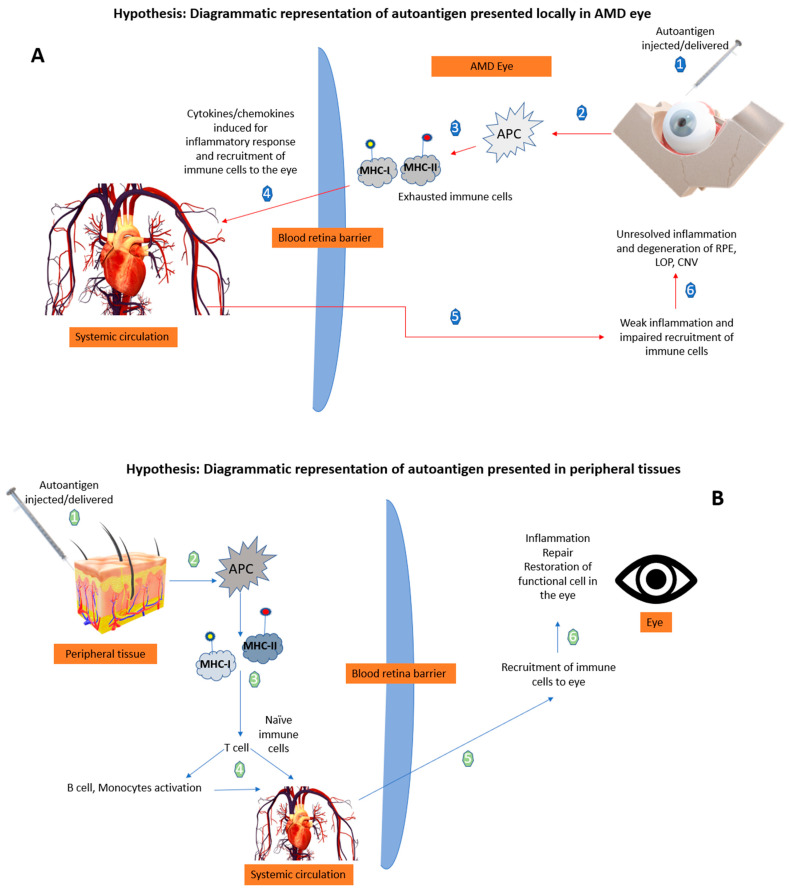
Hypothetical diagrammatic representation of the effects of autoantigen when presented in the AMD eye *versus* peripheral tissues. (**A**) The local delivery of autoantigens in retina is not able to produce a beneficial response by recruiting immune cells to the site (numbers 1–6 explains the sequence of mechanisms when autoantigen is delivered in the AMD eye). (**B**) The peripheral tissue delivery of autoantigens evokes a local beneficial response resulting in recruitment of immune cells to the retina mediated by T cells (numbers 1–6 explains the sequence of mechanisms when autoantigen is delivered in the peripheral tissues) APCs: antigen presenting cells; MHC: major histocompatibility complex; Ap: antigen presentation; RPE: retinal pigment epithelium; LOP: loss of photoreceptors; CNV: choroidal neovascularization; arrow: blue represents the peripheral route, and red represents the local ocular route.

Figure 2 illustrates that in AMD, already exhausted immune cells presenting the retinal self-antigen might generate a locally harmful rather than a favorable immune response. We hypothesize that, when these same antigens are presented in peripheral tissues away from the chronic inflammatory site (the AMD eye), a beneficial immune response ensues so that naïve T cells are activated by the APCs. It is noted that the context of the peripheral antigen presentation is a big factor, as this could influence the risk of autoimmune inflammation [51]. Therefore, in the case of AMD, a well-controlled robust peripheral immune response is vital and enables the recruitment of fresh immune cells to the site of injury/ocular-immune crosstalk that can alter the ongoing pathology. It is suggested that the presentation of autoantigens in the periphery in the form of a vaccine to induce such a beneficial immune response could be of therapeutic benefit (Figure 2).

#### Therapeutic Delivery of Autoantigens

**Figure 3:**
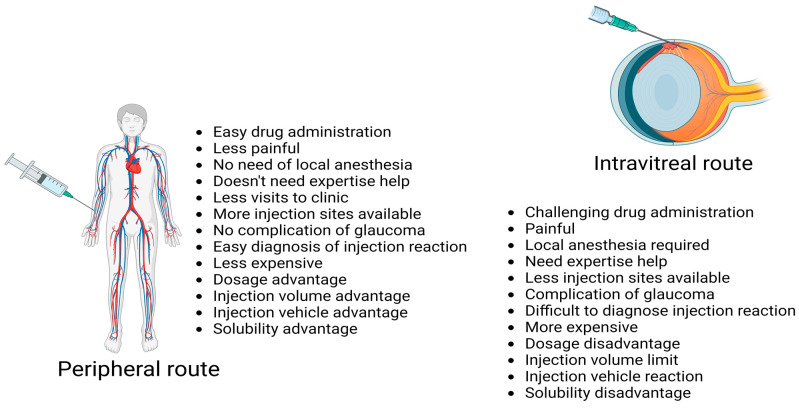
Advantages of peripheral route over intravitreal injection for delivery of drugs. Image created in Biorender.com [71,72,73].

Autoantigen delivery via a site away from injury in age-related neurodegenerative chronic inflammatory disease (ANCID) may be better than one close to the site of injury, both in terms of feasibility and to achieve a better immune response in ANCID like wAMD. There are number of advantages exploiting the peripheral route rather than the intravitreal route (Figure 3).

Antigens are foreign substances that induce an immune response. As they enter our body, immune cells recognize and process them to generate an effective combative response in order to maintain a safe environment for the host. If autoantigens are delivered to peripheral tissues away from the retina and choroid, this increases the possibility of inducing a beneficial immune response that would be absent if autoantigens were delivered to the retina under hostile conditions. The peripheral response could be therapeutic, as it will bring an improved immune balance by recruiting cells from the systemic circulation to the local site of damage (retina) to assist in modifying the ongoing local neurodegenerative situation. A similar approach taken in a previous study has shown that the balance of local versus peripheral immune responses might not be beneficial in every individual, but the benefit can be acquired by boosting/harnessing the response of the systemic immune system [54,74,75]. Bakalash et al. (2005) [74] reported that a similar peripherally mediated process led to the homing of T cells to the eye, and a neuroprotective effect mediated by these recruited T cells was observed in rat models with chronically high intraocular pressure. The above indicates that a boost of the peripheral immune cells may elicit a systemic T cell-mediated response that is beneficial for the repair activity of damaged RPE cells. Table 1 shows the list of autoantigens that have been found from human AMD samples. It is not certain whether all autoantigen peptides can induce a protective effect [54]. A further study might be required to see how immune cells select particular proteins for antigen presentation to elicit protective autoreactive immunity through adaptive immune cells.

## 2. Conclusions

As AMD is a multifactorial disease, a treatment strategy should not focus on a single modifying factor or a single aspect of the inflammatory pathway. Rather, it should encompass common diverse immunological pathways in order to escalate the natural repair processes through the transient expression of effector cells that could modify the disease progression.

## Figures and Tables

**Table 1:** The autoantigens associated with age-related macular degeneration.

No	Autoantigens in AMD	References
1	Cardiolipin	[70]
2	Retinol binding protein (RBP3)	[9]
3	Aldolase C (ALDOC),	[9]
4	Retinaldehyde binding protein 1 (RLBP1)	[9]
5	Pyruvate kinase isozyme M2 (PKM2)	[9]
6	Carboxyethyl pyrrole (CEP)	[63]
7	Annexin A5	[62]
8	HSPA8	[62]
9	HSPA9	[62]
10	HSPB4/CRYAA (Alpha-A Crystallin)	[62]
11	Protein S100-A9/calgranulin B	[62]
12	Alpha-enolase	[68]
13	Alpha-crystallin	[68,69]
14	Glial fibrillary acidic protein (GFAP)	[68]
15	CD5L/Apoptosis Inhibitor of macrophage (AIM)	[64]
